# The Great Roundleaf Bat (*Hipposideros armiger*) as a Good Model for Cold-Induced Browning of Intra-Abdominal White Adipose Tissue

**DOI:** 10.1371/journal.pone.0112495

**Published:** 2014-11-13

**Authors:** Yao Wang, Tengteng Zhu, Shanshan Ke, Na Fang, David M. Irwin, Ming Lei, Junpeng Zhang, Huizhen Shi, Shuyi Zhang, Zhe Wang

**Affiliations:** 1 Institute of Molecular Ecology and Evolution, East China Normal University, Shanghai, China; 2 Department of Laboratory Medicine and Pathobiology, University of Toronto, Toronto, Canada; Albert Einstein College of Medicine, United States of America

## Abstract

**Background:**

Inducing beige fat from white adipose tissue (WAT) is considered to be a shortcut to weight loss and increasingly becoming a key area in research into treatments for obesity and related diseases. However, currently, animal models of beige fat are restricted to rodents, where subcutaneous adipose tissue (sWAT, benign WAT) is more liable to develop into the beige fat under specific activators than the intra-abdominal adipose tissue (aWAT, malignant WAT) that is the major source of obesity related diseases in humans.

**Methods:**

Here we induced beige fat by cold exposure in two species of bats, the great roundleaf bat (*Hipposideros armiger*) and the rickett's big-footed bat (*Myotis ricketti*), and compared the molecular and morphological changes with those seen in the mouse. Expression of thermogenic genes (*Ucp1* and *Pgc1a*) was measured by RT-qPCR and adipocyte morphology examined by HE staining at three adipose locations, sWAT, aWAT and iBAT (interscapular brown adipose tissue).

**Results:**

Expression of *Ucp1* and *Pgc1a* was significantly upregulated, by 729 and 23 fold, respectively, in aWAT of the great roundleaf bat after exposure to 10°C for 7 days. Adipocyte diameters of WATs became significantly reduced and the white adipocytes became brown-like in morphology. In mice, similar changes were found in the sWAT, but much lower amounts of changes in aWAT were seen. Interestingly, the rickett's big-footed bat did not show such a tendency in beige fat.

**Conclusions:**

The great roundleaf bat is potentially a good animal model for human aWAT browning research. Combined with rodent models, this model should be helpful for finding therapies for reducing harmful aWAT in humans.

## Introduction

Obesity is not only a disease [1] but also a momentous risk factor for the development of many diseases, such as diabetes, hypertension, cardiovascular diseases, musculoskeletal disorders and some cancers [2], [3], [4], [5]. According to the World Health Organization Report on Obesity, there were 1.9 billion obese or overweight adults world-wide in 2008, and overweight and obesity became the sixth leading risk for global deaths [6]. The excess costs attributed to health-care due to obesity or overweight have been estimated at over 194.3 billion US dollars, and the costs are more than doubling every decade in America [7]. Therefore, obesity not only jeopardizes human health, but also affects societal development due to the slow progression in the development of effective therapies. Simply, the true face of obesity is the accumulation of adipose tissue *in vivo*.

All mammals possess two major types of adipose tissues, white adipose tissue (WAT) and brown adipose tissue [8], [9]. The former tissue contains large and unilocular lipid vacuoles that store redundant chemical energy in the form of fatty acids [10]. In contrast, the second tissue is characterized by a multilocular lipid vacuole morphology, large numbers of mitochondria and the expression of high levels of *Ucp1*, resulting in a tissue that produces heat by burning lipid in the process of non-shivering thermogenesis [11], [12].

Recently, a third type of adipose tissue, named beige or brite fat, has been identified [13]. Beige fat can be induced from white adipose tissue under specific conditions such as exposure to cold temperatures or by stimulation with β-adrenergic factors [14], [15]. Adipocytes in beige fat have similar characteristics to those in BAT, being defined by the appearance of multilocular lipid droplets, high mitochondrial content and high levels of expression of *Ucp1* when activated [16]. Transformation of beige cells from WAT is significantly enhanced during adaptation to cold temperatures by *Ucp1*-dependent nonshivering thermogenesis [17]. A striking difference between beige fat and BAT is that brown fat cells express high levels of *Ucp1* and *Pgc1a* under the basal state, whereas beige cells highly express these genes only when stimulated, and have near undetectable levels of expression of these genes in the basal state [14], [18].

Although generated by exposure to the same stimuli, the location of WAT has a great effect on the probability of browning. sWAT possesses great plasticity when treated with cold temperatures or β3-adrenergic agonists in rodents [19]. In contrast, when aWAT is exposed to cold temperatures they increase their numbers of lipid droplets in their cells [20]. Moreover, obesity due to sWAT seems to be benign compared to aWAT obesity, which is associated with metabolic diseases in human [21]. aWAT obesity is associated with many diseases, including coronary artery disease, Barrett's esophagus, and type II diabetes [22], [23], [24].

Based on the above, a novel concept of inducing beige fat from the WAT for the treatment of obesity and metabolic disorders is increasingly receiving attention [12], [25], [26]. In the C57BL/6 mouse, white adipose tissue can be induced to make a transition into beige fat by exposure to a cold temperature of 4°C for 1 week [25]. In a thermoneutral environment, *Ucp1*-ablated mice gain weight compared to wild-type mice, whether on normal or high-fat diets, suggesting that the Ucp1 protein has an influence on the development of obesity [27]. *Prdm16* transgenic mice show elevated levels of energy expenditure and limited weight gain in response to a high-fat diet, suggesting that this may be a favorable target for enhancing energy expenditure as an approach to treat obesity [28], [29].

Since beige cells were discovered in 2010 [9], [15], all research concerning the transformation to beige adipocytes has been carried out in rodent models, i.e. mice and rats. While these studies have provided valuable information, their value to the understanding of the human disease is dependent on the equivalence of the rodent models. However, rodent models are likely restricted in their application to humans as beige adipocytes tend to be induced from sWAT, whereas the aWAT is the major risk factor for metabolic diseases in human [30], [31] and human aWAT probably tends to brown after cold acclimation [32], [33]. These two types of WAT, sWAT and aWAT, show different characteristics that may influence the adipose browning process [34].

Bats belong to the second largest order, in number of species, of mammals and have evolved the ability to hibernate. In preparation to hibernate, bats need to store a large amount of fat in their bodies, resulting in body mass increases of 30%–50% compared with non-hibernating conditions, and thus could be considered as being obese [35]. After hibernation, their body weights are significantly reduced. Besides adapting to a cold environment, the excessive fat is used in periods when the bats occasionally awake during the hibernation period and for the arousal from hibernation by BAT non-shivering thermogenesis [36]. Therefore, we assumed that bats might be more likely to develop beige adipocytes, compared to rodents, under exposure to cold temperatures. In this study, we induced beige fat in two bat species, the great roundleaf bat (*Hipposideros armiger*) and the rickett's big-footed bat (*Myotis ricketti*), both of which hibernate, but have differing roosting behaviors and are distantly related [13]. We compared the browning abilities of sWAT and aWAT among the two bat species and mice with the aim to identifying a new animal model for beige fat research.

## Materials and Methods

### Ethics Statement

The field studies did not involve endangered or protected species. This study involving animals strictly followed the Guidelines and Regulations for the Administration of Laboratory Animals (Decree No. 2, the State Science and Technology Commission of the People's Republic of China, November 14, 1998) and were approved by the Animal Ethics Committee of East China Normal University (ID no. AR2012/03001). Bats (Hipposideros armiger and Myotis ricketti) were captured in a cave, named Yulong, in Anhui province and Fangshan Cave in Beijing of China, from May to June 2012. Bats captured in these locations were permitted by Cave Yulong Tourism Development Co., Ltd and Fangshan Bat Protection and Research Center.

### Animals

**Figure 1:**
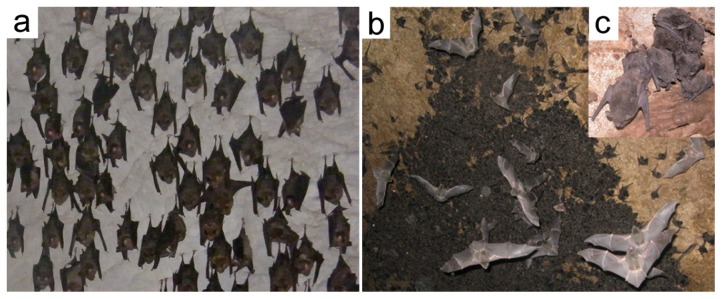
Roosting of great roundleaf bats and rickett's big-footed bats in the wild. (**a**) Great roundleaf bats hung in caves by their foot claws. (**b**) Ricketts big-footed bats crowded in the caves. (**c**) An enlarged view of a group of the Ricketts big-footed bats. They attached their entire ventral surface of their bodies to the rock surface or to another individual's back.

Adult great roundleaf bats (*H. armiger*) were captured from Yulong Cave in Anhui province, China (30°20′N, 117°50′E), and adult rickett's big-footed bats (*M. ricketti*) were captured from Fangshan Cave in Beijing, China (39°42′N, 115°43′E; Figure 1). Bats were immediately transported to the laboratory and housed in a large pet cage (150 cm×180 cm×200 cm) with free access to fresh mealworm (Larval *Tenebrio molitor*) and water. Outbred mice ICR were obtained from SLAC Laboratory Animal (Shanghai, China).

### Temperature Acclimation

Experiments were not carried out until the bats had acclimatized to the local surroundings (25°C). After acclimatization, the great roundleaf bats were divided into seven groups of three individuals of random gender: three groups each for exposure to 15°C and 10°C for periods of 3 days (d), 7 d and 14 d (total 6 groups), and a control group at 30°C for 7 d. The rickett's big-footed bats were divided into three groups of three individuals of random gender: two at 10°C for 7 d and 14 d, and the control at 30°C for 7 d. All temperature acclimation experiments were conducted in an artificial climate incubator (RXZ-128A, Ningbo, China) with 80% humidity. Before exposure to 15°C, bats were allowed to adapt to 20°C for 12 h. Similarly, bats were adapted to 20°C and 15°C for 12 h periods sequentially before exposure to 10°C. Bats were directly exposed to 30°C from room temperature (25°C). At room temperature, bats, of each species, were kept in one cage, while at 10°C, 15°C and 30°C, pairs of bats were kept in a single cage.

ICR mice (6∼9 weeks old) were housed for at least two weeks at room temperature after arrival, and then they were divided into five groups of three individuals of random gender: two groups were at 15°C with exposure for 7 d and 14 d, two at 10°C for 7 d and 14 d, and one at 30°C for 7 d as a control. Mice were kept in an artificial climate chamber (RXZ-128A, Ningbo, China) with 60% humidity with 12-hour light and 12-hour dark cycles, and had free access to chow and water. Mice were individually kept in cages.

### Sample Collection

**Figure 2:**
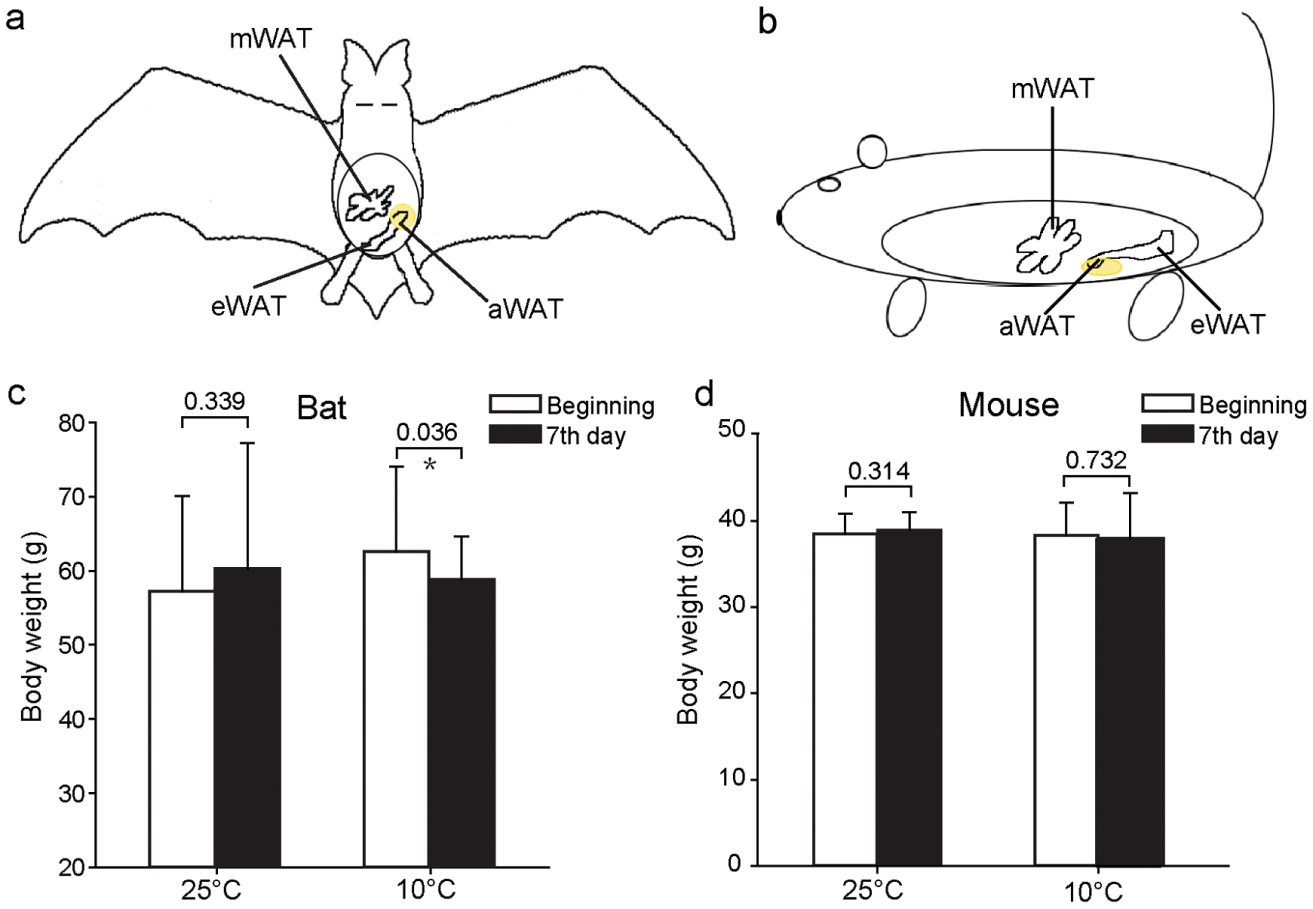
Schematic diagram of intra-abdominal adipose tissue (aWAT), and changes in body weight of bats and mice after exposure to cold temperatures. (a–b) Schematic diagram of aWAT in the bat (a) and mouse (b). Tissues depicted in yellow are aWAT studied here. mWAT: mesenteric WAT; eWAT: epididymal WAT. (c–d) Changes in the body weighs of bats (c) and mice (d), where one group was kept at room temperature (25°C) and the other at 10°C. Each group was composed of 6 individuals. Data are expressed as means ± SD. Statistical significance is marked above the bars: *, p<0.05.

After temperature acclimation, bats and mice were euthanized by cervical dislocation. Adipose tissues were divided into two sides (left and right). One side was used for RNA extraction, and the other was fixed in 4% paraformaldehyde (PFA, Sigma-Aldrich, St. Louis, MO, USA) for morphological study. Three types of adipose tissues were collected (iBAT, sWAT and aWAT). Depot of the classical interscapular brown adipose tissue (iBAT) is the largest BAT reservoir in mammals. Subcutaneous adipose tissue (sWAT) was collected from adipocytes beneath the skin and above the abdominal wall in bats and from the inguen in the mice. Intra-abdominal white adipose tissue (aWAT) was collected from the fat tissue between the abdominal wall and the viscera, which includes some epididymal WAT but excludes mesenteric WAT (Figure 2a and b) [37].

### Morphological Study

Adipose tissues were fixed in 4% PFA overnight and then thoroughly washed in PBS. The tissues were then dehydrated in a series of graded ethanol, cleared in xylene and then embedded in paraffin. Adipose tissues were sectioned to 5 µm slides by a microtome (Leica RM2235, Germany), stained in haematoxylin and eosin (HE staining), and photographed under a microscope (Leica DM 2500, Germany). HE staining photos from three individuals were used for calculation of adipocyte diameters and the diameters were calculated by Image J software (http://rsbweb.nih.gov/ij/).

### Real Time Quantitative PCR (RT-qPCR)

Total RNA was extracted from fresh adipose tissue using the RNeasy Lipid Tissue Mini Kit (Qiagen, Hilden, Germany) according the handbook, and was reverse transcribed using the ABI high-capacity cDNA reverse transcription kit (Applied Biosystems, Foster City, USA). SYBR Premix Ex Taq (TaKaRa, Dalian, China) was used to determine transcript levels and was carried out on an ABI/Prism 7300 instrument (Applied Biosystems, USA) by RT-qPCR technology. Changes in *Ucp1* and *Pgc1a* expression levels in WAT were used to distinguish beige fat from brown fat after cold acclimation [16]. The mean value of the sample duplicates were normalized to that of the TATA box-binding protein (*Tbp*) gene using a comparative (2^-ΔΔCt^) method [38]. RT-qPCR primers for the bats and mice were listed in Table 1.

**Table 1:** Sequences of primer used for RT-qPCR.

Species	Gene	Forward primer (5' → 3')	Reverse primer (5' → 3')	Product (bp)
*H. armiger*	*Ucp1*	GGAACAATCATCACTCTGGC	CCTAAACTACTTTCTTTCCCTG	158
*H. armiger*	*Tbp*	GGACCACCGCCCTGATATT	AGCCCAGCTTCTGAACGACT	115
*H. armiger*	*Pgc1a*	GCTGAAGAGGGAAGAATACCG	TACCAACATAAATCACACGGCG	116
*M. ricketti*	*Ucp1*	AAGGTGAATGCCCGAACTCC	TAGTTTCTCTGCCCTCGGTGA	198
*M. ricketti*	*Tbp*	ATTCAGAACATGGTGGGGAGC	CAGGAAATAGCTCTGGCTCGTAA	106
*M. ricketti*	*Pgc1a*	CTGACCACAAACGATGACCCTC	TTGGCTTGTAAATGTTGCGACT	122
*M. musculus* *	*Ucp1*	CTGCCAGGACAGTACCCAAG	TCAGCTGTTCAAAGCACACA	112
*M. musculus*	*Tbp*	GCTGTAAACTTGACCTAAAGACCAT	AACGCAGTTGTCCGTGGCTCT	147
*M. musculus* *	*Pgc1a*	CCCTGCCATTGTTAAGACC	TGCTGCTGTTCCTGTTTTC	161

* Primers for mice were from a previous publication [17].

## Results

### Distinct Roosting Behaviors of the Great Roundleaf Bat and the Rickett's Big-footed Bat

In the wild, the great roundleaf bats and the rickett's big-footed bats display very different roosting behaviors in the caves in the non-hibernating season, when these individuals were caught (Figure 1). When hanging on the rock and resting, the great roundleaf bats are vigilant to the environment and kept moving their ears to listen for sounds from their surroundings. The great roundleaf bats hung from rock surface only by their foot claws, and never attached their bodies to rock surfaces or other individuals, keeping a distance of about 5 cm from each other (Figure 1a). If one bat is accidently touched by another, then they would fight for space. In contrast, the rickett's big-footed bats attach their entire ventral surface of their bodies to the rock surface or to another individual's back and make crowed communities in the caves (Figures 1b and c).

During cold acclimation in the lab, the great roundleaf bats remained vigilant to their surroundings and did not enter hibernation due to lack of food or water supply and it was not prevented by the occasionally disturbance. However, rickett's big-footed bats crowded into a corner of the cage and disliked moving or eating in response to cold. Finally, these bats stopped taking food and started hibernation on the fifth day of exposure to 10°C temperatures.

### More Severe Morphological Changes in aWAT Adipocytes of the Great Roundleaf Bat than those in the Mouse

**Figure 3:**
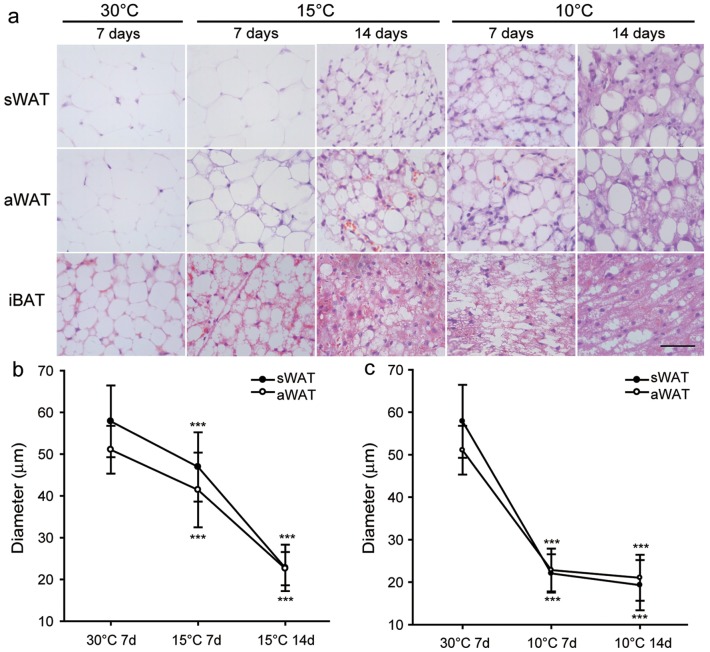
Morphological observations in the great roundleaf bats. (**a**) HE staining. (**b–c**) Measurement of adipocytes diameters of sWAT and aWAT from the cold and 30°C temperature groups. Diameter is expressed as means ± SD. Statistical significance: *, p<0.05; **, p<0.01; ***, p<0.001.

**Figure 4:**
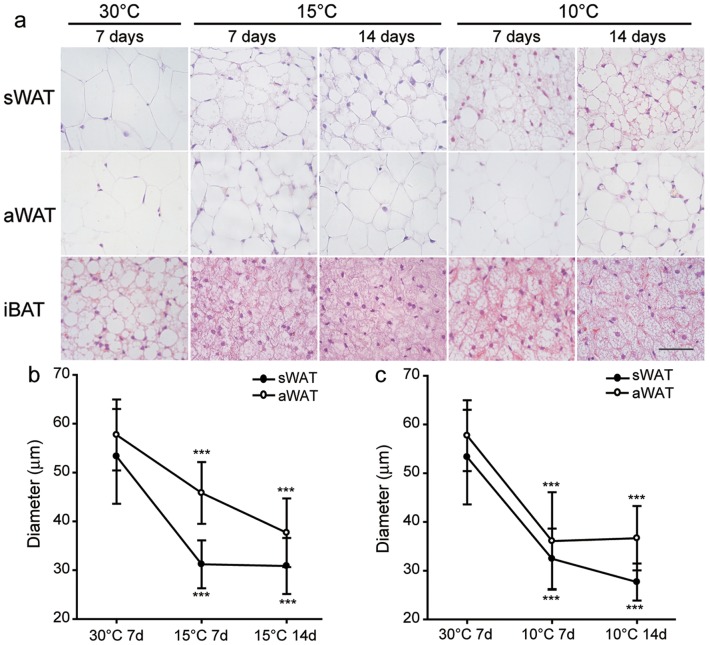
Morphological observations in mice. (**a**) HE staining. (**b–c**) Measurement of adipocytes diameters of sWAT and aWAT from the cold and 30°C temperature groups. Diameter is expressed as means ± SD. Statistical significance: *, p<0.05; **, p<0.01; ***, p<0.001.

After exposure at 10°C for 7 d, the body weight of the bats was significantly decreased (3.82±3.27 g, p = 0.036) compared to room temperature, while a non-significant change was seen mice (0.3±2.14 g, p = 0.732) (Figure 2c and d). The aWAT and sWAT of the great roundleaf bat became brown-like in morphology after exposure to cold, with the aWAT changed more severely than sWAT at 15°C for 7 d (Figure 3a). Fat droplets in WAT became smaller and were multilocular, and the number of nuclei increased within a unit area of a slide. Moreover, longer exposure times and lower environment temperatures resulted in increases in the browning change of increases in the amount of cytoplasm. In the mouse, aWAT and sWAT also underwent brown-like morphological changes, however, these changes were less dramatic than those in seen the great roundleaf bat, especially the aWAT (Figure 4a). aWAT adipocytes in mice retained unilocular lipid vacuoles, with only slight decreases in size. Although fat droplets in sWAT became multilocular and small, the amount of cytoplasm increased less than that seen in the great roundleaf bat at the corresponding exposure situations.

Measurements of the diameters of sWAT and aWAT adipocytes were in accordance with the morphological observations. Compared with exposure to 30°C, the diameters of the white adipocytes in both the great roundleaf bat and the mouse were significantly decreased (P<0.001) after exposure to 15°C or 10°C for at least 7 days. In bats, the diameters of the aWAT and sWAT adipocytes decreased to 23 µm for both after exposure to 15°C for 14 d, 22 µm and 23 µm after 10°C for 7 d, 21 µm and 19 µm after 10°C for 14 d, compared to the diameters of 51 µm and 58 µm after 30°C for 7 d (Figures 3b and c). In the mouse, the diameters of the aWAT and sWAT adipocytes dropped to 38 µm and 31 µm after exposure to 15°C for 14 d, to 36 µm and 32 µm after 10°C for 7 d, and to 36 µm and 28 µm after 10°C for 14 d, compared with 58 µm and 53 µm after 30°C for 7 d (Figures 4b and c). The original diameters of the aWAT and sWAT adipocytes in both bat and mice were between 50 and 60 µm at 30°C, but upon exposure to cold temperatures, bats yielded the smallest diameters of around 20 µm, while in mice they only decreased to about 30 µm.

### Significantly Increased Expression of *Ucp1* and *Pgc1a* in Beige Fat of the Great Roundleaf Bat

**Figure 5:**
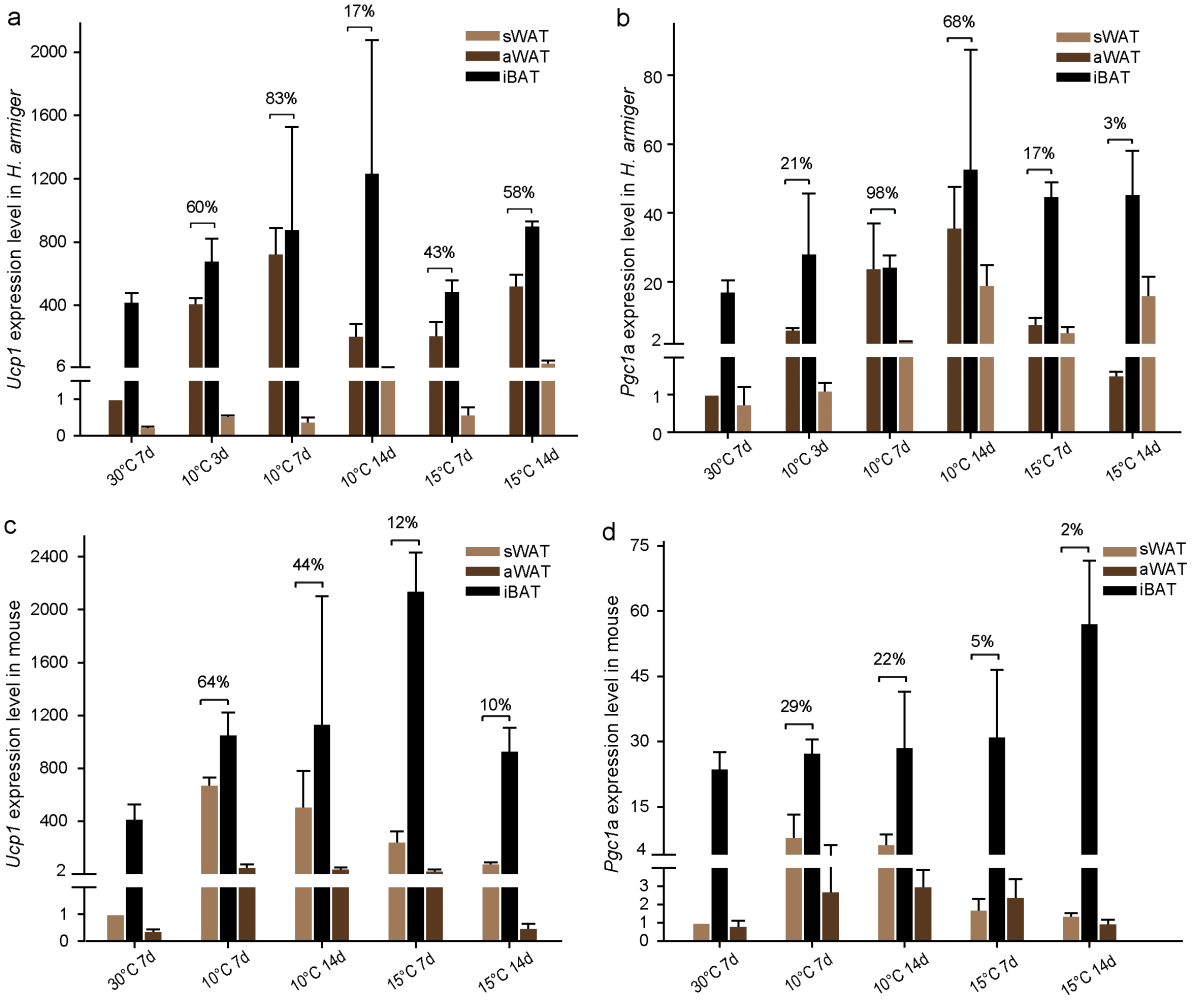
Comparison of relative expression levels of *Ucp1* and *Pgc1a* between WATs and BAT. (**a–b**) *Ucp1* (**a**) and *Pgc1a* (**b**) expression levels in aWAT, iBAT and sWAT of *H. armiger* after cold acclimation relative to the levels in aWAT exposed to 30°C for 7 days. (**c–d**) *Ucp1* (**c**) and *Pgc1a* (**d**) expression levels in sWAT, iBAT and aWAT of mice relative to the levels in sWAT exposed to 30°C for 7 days.

Relative expression levels for *Ucp1* and *Pgc1a* in adipose tissues were estimated using RT-qPCR. In the great roundleaf bat, *Ucp1* expression was significantly increased by 729 fold in aWAT (P<0.01) and 241 fold in sWAT (P<0.01) at the highest levels after exposure to cold compared with the 30°C control (Figure 5a). *Pgc1a* expression increased significantly 36 fold in aWAT (P<0.01) and 29 fold in sWAT (P<0.01) at the highest levels (Figure 5b). In contrast, expression of *Ucp1* and *Pgc1a* in iBAT showed relatively similar levels after exposure to cold compared to the 30°C control (Figures 5a and b). In the mouse, *Ucp1* expression increased significantly by 139 fold in aWAT (P<0.01) and 676 fold in sWAT (P<0.01) at the highest levels after cold exposure compared to the 30°C control (Figure 5c). *Pgc1a* expression increased significantly 4 fold in aWAT (P<0.01) and 8 fold in sWAT (P<0.05) at the highest levels (Figure 5d). As in the bat, expression of *Ucp1* and *Pgc1a* in iBAT showed relatively similar levels after exposure to cold compared to the 30°C control (Figures 5c and d).

### Differing Degrees of Browning of aWAT and sWAT in the Great Roundleaf Bat and the Mouse

An interesting observation is that in the great roundleaf bat the degree of browning of aWAT was greater than that for sWAT, whereas in the mouse the pattern reversed, with the degree of browning of sWAT being greater than that for aWAT, when assessed comparing the levels of expression of *Ucp1* and *Pgc1a* in WATs to those in iBAT (Figure 5). In the great roundleaf bat, expression of the two genes in the aWAT-originated beige fat increased to 83% and 98%, at their highest percentages, of the levels seen in iBAT after exposure to 10°C for 7 days, suggesting the aWAT was almost fully stimulated (Figures 5a and b). The highest levels of expression for these genes in sWAT-originated beige fat took longer exposure (10°C exposure for 14 days) and were lower than those in the fully stimulated aWAT. In the mouse, expression of *Ucp1* and *Pgc1a* in sWAT-originated beige fat increased to 64% and 29%, at their highest percentages, of the levels seen in iBAT after exposure to 10°C for 7 days, and they were higher than those in aWAT-originated beige fat, which were relatively stable compared to the amounts found in iBAT (Figures 5c and d).

### Difficulty in Inducing Beige Fat by Cold Temperature Exposure in the Rickett's Big-footed Bat

**Figure 6:**
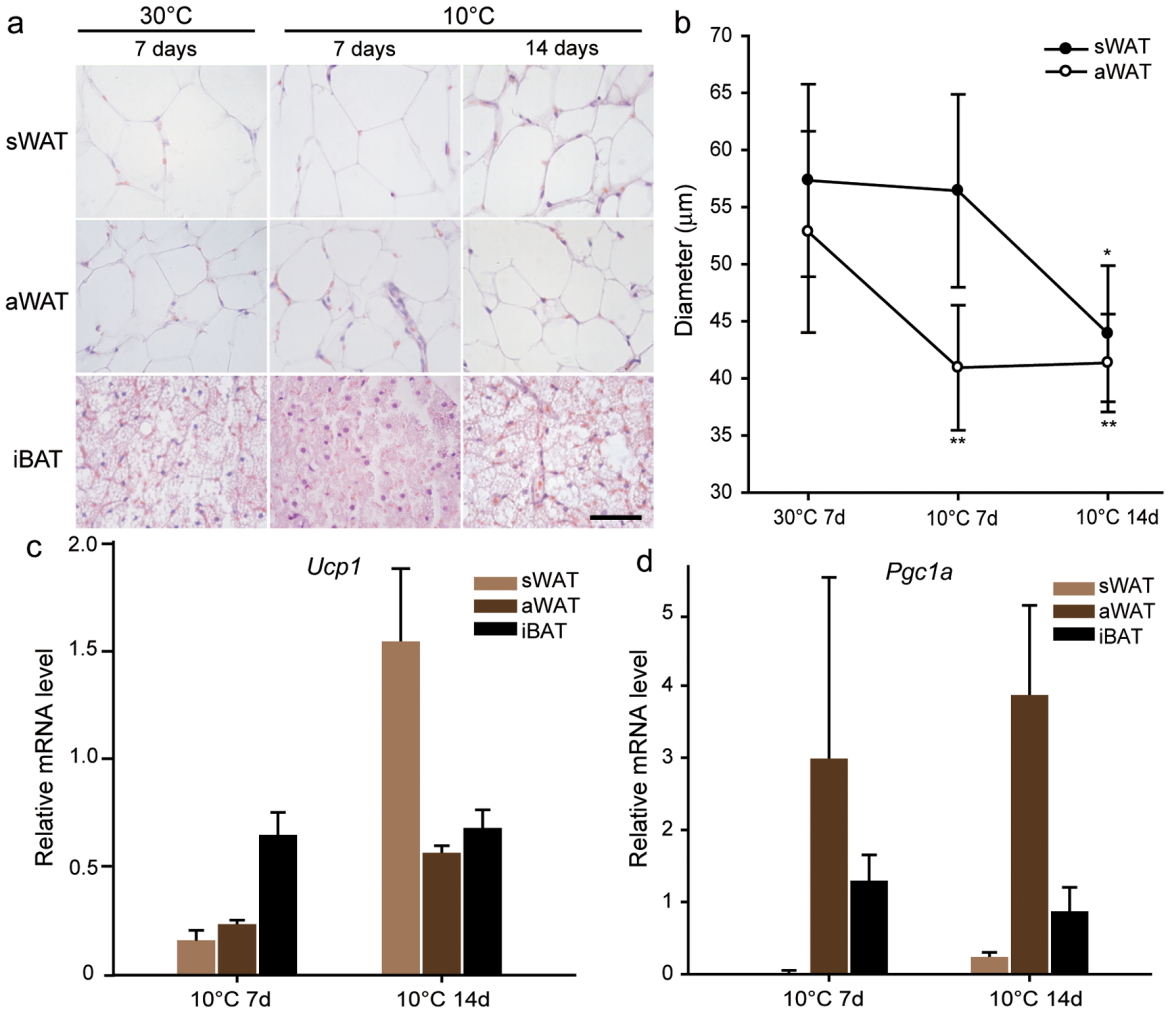
Morphological observations and relative expression levels of *Ucp1* and *Pgc1a* in the rickett's big-footed bats. (**a**) HE staining. (**b**) Measurement of adipocytes diameters of sWAT and aWAT from 10°C and 30°C temperature groups. (**c–d**) Relative expression levels of *Ucp1* (**c**) and *Pgc1a* (**d**) in sWAT, aWAT and iBAT after cold acclimation, comparing to the 30°C control of the corresponding tissue. Fold change expressed as means ± SD. Statistical significance: *, p<0.05; **, p<0.01.

In cold temperatures the rickett's big-footed bats were observed to attach to each other tightly in the corner of the cage, where they would eat few mealworms and drink little water. The rickett's big-footed bats stopped taking food and water and fully went into hibernation, with relative low body temperature, on the fifth day of exposure at 10°C. In contrast to the great roundleaf bat, very few morphological changes were seen in the adipocytes from the rickett's big-footed bat after cold acclimations (Figure 6a). WAT adipocytes retained the appearance of unilocular lipid droplets with peripheral nuclei, and iBAT had multilocular droplets with central nuclei. Although the diameters of the WAT adipocytes decreased after exposure to cold, the decrease in diameter was only to 41 µm, compared to the smallest diameter of 19 µm seen in the great roundleaf bat (Figures 6b). In agreement with the morphological data, expression of *Ucp1* and *Pgc1a* was not significantly increased after exposure to 10°C for 7 or 14 days (Figures 6c and d).

## Discussion

Previous studies have indicated that intra-abdominal adiposity is strongly associated with increased mortality and many metabolic diseases [31]. People having an inactive lifestyle preferentially increase visceral adipose tissue and get ill [39]. Rodents, the traditional animal model, have provided us a great number of insights into knowledge concerning beige fat and promising therapies for the treatment of obesity and related diseases [16], [40]. However, the differing recruitment of beige fat from sWAT and aWAT in rodents compared to humans suggests that the rodent models may be more useful for the study of sWAT browning, whereas aWAT browning is apparently more important for human health [32], [33]. Here, we describe a new mammalian model, the great roundleaf bat, which can easily be induced to produce beige fat by exposure to cool (15°C) or cold (10°C) temperatures for a relative short period of time (3–14 days). Both sWAT and aWAT from this bat show significantly increased levels of *Ucp1* and *Pgc1a* expression after exposure to cold temperatures, and the morphology of their adipocytes showed brown-like characters. The concordance of the thermogenic gene expression profiles and morphological peculiarities strongly suggest that beige fat was induced in the great roundleaf bat. More importantly, aWAT browning is rapid and much more pronounced than sWAT browning in the great roundleaf bat, which is the opposite pattern to the browning seen in rodents [40].

Our morphological studies and the gene expression data show that the degree of browning of WATs was associated with temperature and exposure time in both the great roundleaf bat and the mouse. While little morphological change was observed in the multiocular cells, elevated expression of brown-fat-cell-associated genes, such as *Ucp1* and *Pgc-1a*, in the white adipose tissue suggests that they are undergoing browning. Longer periods of cold exposure and lower temperatures resulted in smaller white adipocytes in both species. Adipose cell size is inversely related to adiponectin levels [41]. Leptin levels are known to increase in obese humans and fall after weight loss [42]. Other factors, such as interleukin-1 (IL-1), interleukin-6 (IL-6) and interleukin-10 (IL-10), are associated with inflammation and are elevated in obese human [43]. These factors deserve further study in our bat model. However, *Ucp1* expression in aWAT beige cells reached their peak levels after exposure to 10°C for 7d in the great roundleaf bat, and then dropped. In the mouse, this and previous studies showed a similar expression patterns for *Ucp1* in sWAT [25], [44]. Moreover, the great roundleaf bat needed a longer period of time to reach peak expression levels for *Ucp1* when exposed to a 15°C temperature, and its sWAT needed a longer time to become beige fat than the aWAT. These results suggest that lower temperatures generated greater stimulation on WATs, and that more mild cold exposure requires more prolonged exposure to induce a similar effect. These results also suggest that a particular time point should be identified for each animal model to identify the best efficiency for producing beige cells.

Beige adipocytes emerge from sWAT exposed to cold or other stimulus in mice and express high levels of thermogenic genes [14], [17], [45], [46]. Although aWAT-originated beige fat also expresses these genes, the levels were much lower than those from sWAT-originated beige fat [37]. sWAT that is located beneath the skin is capable of reacting directly to the ambient temperature, while aWAT, which located in the abdomen, experiences the environment indirectly. This fact was considered to be responsible for the priority of sWAT browning in mice [47]. In contrast, we found that aWAT browning was earlier and more extensive than sWAT browning in the great roundleaf bat. Moreover, fully simulated aWAT-originated beige fat expressed levels of *Ucp1* comparable to those of classic BAT. These new results indicate that the order and extent of browning in different fat depots is highly variable among mammalian species.

It is worth noting that not all bat species are suitable for inducing beige fat. In this study, we found that a different bat species, the rickett's big-footed bat, is such the case. The browning phenomena in the rickett's big-footed bat differed from that seen in the great roundleaf bat. These two bat species belong to separate suborders of Chiroptera and possess very different roosting behavior [48]. Great roundleaf bats keep a distance from each other, while the rickett's big-footed bats tend to attach to each other during hibernation. This difference of habit likely explains at least part of the difference of browning in these two species. The great roundleaf bat would naturally need to produce more heat by itself, compared to the rickett's big-footed bat. The long evolutionary distance between the species and their behavioral differences might explain their differing abilities in WAT browning.

In conclusion, we suggest that the great roundleaf bat could be a useful animal model for beige fat related studies as it has the following advantages. First, fully stimulated beige fat can be induced under relatively warm temperatures and short periods of time. Second, this species is widely distributed in South Asia and is easy to obtain and maintain. Third, the genome and transcriptome of this species is currently being sequencing (unpublished results), which should provide extensive genomic information for this new animal model. Lastly, this appears to be the best animal model found that replicates human aWAT browning. We believe that additional closely related bat species with similar roosting behaviors to those of the great roundleaf bat might also be used for aWAT browning research. Besides *Ucp1* and *Pgc1a*, changes in the expression of adipokines and other factors in cold-induced bats should be interesting to investigate in future studies.
